# Carbon Storage Change Analysis and Emission Reduction Suggestions under Land Use Transition: A Case Study of Henan Province, China

**DOI:** 10.3390/ijerph18041844

**Published:** 2021-02-14

**Authors:** Dongyang Xiao, Haipeng Niu, Jin Guo, Suxia Zhao, Liangxin Fan

**Affiliations:** 1 School of Surveying and Land Information Engineering of Henan Polytechnic University, Jiaozuo 454000, China; 111704010001@home.hpu.edu.cn (D.X.); 111904010001@home.hpu.edu.cn (J.G.); zhaosuxiahpu@hpu.edu.cn (S.Z.); flx@hpu.edu.cn (L.F.); 2 Research Centre of Arable Land Protection and Urban-Rural High-Quality Development of Yellow River Basin, Henan Polytechnic University, Jiaozuo 454000, China

**Keywords:** carbon emissions, LUCC, ecological compensation, multi-river basins, Henan province

## Abstract

The significant spatial heterogeneity among river basin ecosystems makes it difficult for local governments to carry out comprehensive governance for different river basins in a special administrative region spanning multi-river basins. However, there are few studies on the construction of a comprehensive governance mechanism for multi-river basins at the provincial level. To fill this gap, this paper took Henan Province of China, which straddles four river basins, as the study region. The chord diagram, overlay analysis, and carbon emission models were applied to the remote sensing data of land use to analyze the temporal and spatial patterns of carbon storage caused by land-use changes in Henan Province from 1990 to 2018 to reflect the heterogeneity of the contribution of the four basins to human activities and economic development. The results revealed that food security land in the four basins decreased, while production and living land increased. Ecological conservation land was increased over time in the Yangtze River Basin. In addition, the conversion from food security land to production and living land was the common characteristic for the four basins. Carbon emission in Henan increased from 134.46 million tons in 1990 to 553.58 million tons in 2018, while its carbon absorption was relatively stable (1.67–1.69 million tons between 1990 and 2018). The carbon emitted in the Huai River Basin was the main contributor to Henan Province’s total carbon emission. The carbon absorption in Yellow River Basin and Yangtze River Basin had an obvious spatial agglomeration effect. Finally, considering the current need of land spatial planning in China and the goal of carbon neutrality by 2060 set by the Chinese government, we suggested that carbon sequestration capacity should be further strengthened in Yellow River Basin and Yangtze River Basin based on their respective ecological resource advantages. For future development in Hai River Basin and Huai River Basin, coordinating the spatial allocation of urban scale and urban green space to build an ecological city is a key direction to embark upon.

## 1. Introduction

Human social and economic activities, which ultimately are manifest in changed land-use space [1,2,3], usually entail massive energy consumption [4]. The chain reaction caused by this energy consumption leads to increased carbon dioxide emissions and climate warming. Artificial land use/cover change (LUCC), therefore, plays an important role in affecting the dynamics of ongoing global warming and the carbon cycle [5]. Evidence has shown that carbon emissions caused by LUCC accounted for approximately 30% of carbon emissions caused by human activities since the Industrial Revolution [6,7].

Given the significant differences in carbon emissions caused by various land-use types, LUCC may lead to alterations in carbon emissions and carbon absorption among certain regions [8]. There is no doubt that the spatial-temporal trajectories of carbon emissions under the disturbance of LUCC have been confirmed by a huge body of research. Farmland, which contributes 7–20% of the world’s total greenhouse gas emissions [9], is a proven contributor to carbon emissions. In particular, paddy fields are generally considered to be significant contributors to net carbon emissions [10]. Forest ecosystems are one of the largest terrestrial ecosystem carbon pools, and the carbon storage of forest ecosystems accounts for 50–60% of the world’s carbon storage [11]; hence, the carbon storage potential of forests has motivated the research interest of many scientists [12,13,14]. Construction land is deemed a major carbon source [15], because it portends substantial energy consumption which will generate massive carbon emissions [16,17,18]. In addition, researchers have also focused on the carbon emission effects caused by different ecosystems. For example, evidence revealed that the conversion of woodland to farmland would reduce the land’s carbon sink capacity [19,20]. More recently, some scholars [6,21] analyzed the spatio-temporal characteristics of carbon storage changes caused by LUCC.

China, which has the third largest land area in the world, is also the largest developing country. Its rapid urbanization in recent years has placed tremendous pressure and difficulty upon China’s ability to attain carbon emission reductions in the near future [22]. In the General Debate of the Seventy-fifth UN General Assembly held in September 2020, President Xi Jinping proposed that China would strive to reach peak carbon dioxide emissions by 2030, and soon after achieve carbon neutrality, by 2060. The Chinese government’s determination to reduce carbon dioxide emissions has steered provinces to face new challenges towards developing a low-carbon economy. Henan Province is China’s largest province in terms of agricultural production, home to a population of nearly 100 million people [23]. According to the latest statistics, the total carbon emissions in Henan Province increased by more than 25% from 2006 to 2015 [24]. Many studies have measured the fate of carbon emissions caused by LUCC in Henan province [9,23,24,25], but these tend to focus on carbon emissions within an administrative region at provincial, municipal or county level. This has left the analysis of carbon emission effects caused by LUCC along natural boundaries (such as basin boundaries) generally understudied. As is well known, the river basin is a relatively independent and complete spatial unit demarcated by a natural hydrological system, but the basin space is usually divided into multiple administrative regions. Because of differences in their geographical location, natural conditions, economic technology, and historical background, human activities across different basins are spatial heterogeneous [26,27]. Henan Province, with a land area of ca. 167,000 km^2^, spans four river basins: Hai River Basin, Yellow River Basin, Huai River Basin, and Yangtze River Basin. With the ecological protection and high-quality development of the Yellow River Basin recently upgraded as a national strategy by Chinese government in 2019, Henan Province has since attached great importance to the comprehensive management of river basin ecology.

Therefore, exploring the temporal and spatial trajectory of carbon storage changes as caused by land use changes in Henan Province, from the basin scale, is imperative to provide a timely and accurate baseline reference for low-carbon economic development and the comprehensive management of river basins in Henan Province. Based on this, this paper had two objectives: (1) To propose and implement a new perspective by which to analyze the temporal and spatial characteristics of carbon storage changes caused by LUCC within a province at the basin scale, and (2) to provide decision support for the development of emission reduction strategies for use in the comprehensive management of multi-river basins in Henan Province. To do this, here we used chord diagrams and the overlay analysis function of ArcGIS 10.7 (Esri, Redlands, CA, USA) to analyze the spatio-temporal trajectory of LUCC in the four river basins of Henan Province, from 1990 to 2018. Then, carbon storage changes caused by LUCC in the study area from 1990 to 2018 were estimated based on energy consumption data. Finally, we put forward policy recommendations for the reduction of carbon emissions in the four river basins of Henan Province, based on the principle of land space planning. This paper, on the one hand, fills the gaps in theoretical research on the contradictions and conflicts between human activities and resource protection in these administrative regions which are usually divided by multi-river basins. On the other hand, our work makes a contribution to the comprehensive governance of river basins at the provincial level and have certain reference significance for local governments with similar situations in China and other countries.

## 2. Materials and Methods

### 2.1. Study Area

**Figure 1:**
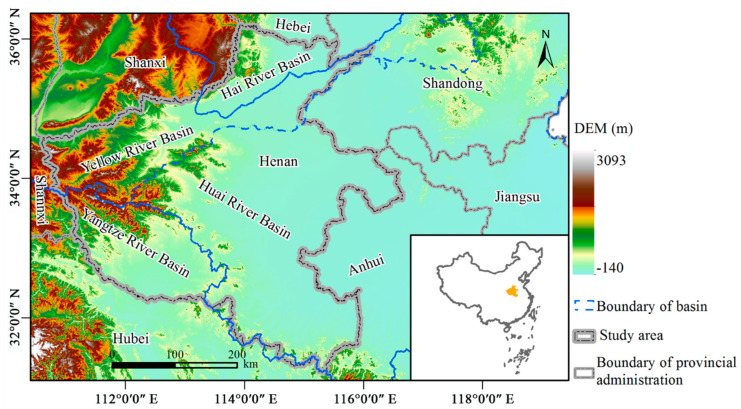
Geographical location of Henan Province in China.

Located in east-central China (Figure 1), Henan Province (110°21′ E–116°39′ E, 31°23′ N–36°22′ N) sustains ca. 96 million people in a land area of nearly 16.7 × 10^4^ km^2^, according to official statistics for 2018. It is one of the 13 major grain-producing areas designated by the Chinese government, due to its significant food and agricultural production capacity [9,23]; in 2018, the grain output exceeded 66 million tons in a total grain-sown area of 10.9 × 10^4^ km^2^. In the past 29 years, the GDP of Henan Province has increased from 14.6 billion USD in 1990 to 752 billion USD in 2018, while the proportion of its area that is urbanized rose from 15.5% to 51.7%. This rapid growth in urbanization and economic output has resulted in great changes in land-use patterns [25] and high emissions of pollutants [9].

As Figure 1 illustrates, Henan Province’s territory is entirely occupied by four basins: Hai River Basin (HRB), Huai River Basin (HURB), Yellow River Basin (YERB), Yangtze River Basin (YARB). Among them, most of the plains are distributed in the HURB, whereas the mountains are concentrated in the YERB and YARB; the land area of the HRB is the smallest. The Yellow River and Yangtze River are the first and second largest rivers in China, respectively; hence, the corresponding regions they flow through in the YERB and YARB have developed rapidly. In particular, since President Xi Jinping proposed “Promoting the ecological protection and quality development of the Yellow River Basin” in 2019, China has integrated the comprehensive management of the YERB into its national strategy. In response to this national call, the government of Henan has strengthened the ecological protection of the four river basins.

### 2.2. Data Sources

The following five data types and sources were used to carry out this study:

(1) Socio-economic development data. The land area, population, GDP, urbanization proportion, and agriculture data related to the study area were all derived from the Henan Statistical Yearbook (2019).

(2) Digital elevation model (DEM) data. The DEM was derived from the SRTMDEMUTM 90M Resolution Digital Elevation Data product, provided by Geospatial Data Cloud Website (http://www.gscloud.cn/, accessed on 21 June 2020).

(3) The administrative boundaries and river basin boundaries were obtained from the Resource and Environment Science and Data Center of the Chinese Academy of Sciences (http://www.resdc.cn/, accessed on 21 June 2020).

(4) Energy consumption data. Data on the usage of coal, coke, gasoline, kerosene, diesel oil, fuel oil, liquefied petroleum gas, and natural gas were derived from China Energy Statistical Yearbook. Since the latest China Energy Statistical Yearbook had been last updated in 2017, we used that year’s energy consumption data for 2018.

(5) Land use remote sensing data. For this, data for the years 1990, 2000, 2010, 2015, and 2018 were provided by the Resource and Environment Science and Data Center of the Chinese Academy of Sciences. The assembly of this dataset, whose overall accuracy is 88.95%, was based on Landsat raster images with a 30-m resolution, for which the 1990, 2000 and 2010 data used Landsat- TM/ETM images, and that of 2015 and 2018 used Landsat-OLI images [28,29]. By referring to land functions and the previous studies [28,30], the original land-use types were reclassified into ecological conservation land, food security land, and production and living land. Specifically, ecological conservation land included woodland, grassland, water bodies, and unused land, because these land-use types have significant ecological service functions. Farmland was regarded as food security land because of its food production function. Production and living land consisted of urban land, rural settlement, and other construction land.

### 2.3. Methods

#### 2.3.1. Land-Use Change Analysis

We completed the analysis of land-use change via three steps: first, using the spatial clipping tool of ArcGIS 10.7 and the vector boundaries of four basins in Henan Province, we obtained land-use vector data for each of the four basins in 1990, 2000, 2010, 2015, and 2018. Second, a land-use transfer matrix was built to calculate the net area of land-use conversion within the four basins during five time-period intervals: 1990–2000, 2000–2010, 2010–2015, 2015–2018, and 1990–2018. Then, the chord diagram, drawn in RStudio software with the ‘Circlize’ package [31], was used to visualize the flow, direction, and diversity of LUCC changes in each specific period. Finally, space trajectories of the LUCC within the four basins were analyzed, using the overlay analysis tool in ArcGIS 10.7, during the five periods encompassing the 1990 to 2018 timeframe.

#### 2.3.2. Computation of Carbon Storage Change Caused by LUCC

According to the published results, changes of carbon storage caused by land-use changes may be divided into carbon absorption and carbon emissions. Woodland, grassland, water and unused land are usually regarded as carbon sinks, because of their ability to absorb carbon [5,32]. Most studies have confirmed that the total amount of CO_2_ and CH_4_ emitted by crops is greater than the amount of CO_2_ absorbed by these plants [33,34,35]. Farmland, therefore, is typically deemed a carbon source. Construction land—here the pooled areas of urban land, rural settlement, and other construction land—is an important carbon source, because the human production and living activities associated with this land use consume a staggering amount of energy [36]. Therefore, here we only calculate the carbon absorption of woodland, grassland, water, and unused land, as well as the carbon emission of farmland and construction land.

(1) Computation of carbon absorption: This can be calculated as follows [5]:

(1) $$E_{a}=\sum e_{i}=\sum T_{i}\cdot \delta_{i}$$

where, $E_{a}$ denotes the carbon absorption; $e_{i}$ is the carbon absorption caused by a certain land-use type; $T_{i}$ is the area of a certain land-use type; and $\delta_{i}$ represents the carbon absorption coefficient of a certain land-use type (Table 1).

(2) Computation of carbon emission: This calculation method and carbon emission coefficient of farmland are respectively shown in Formula (1) and Table 1. The carbon emission of construction land is indirectly estimated by the carbon emission coefficient of energy consumption [35]. Referring to the practical situation in Henan, the energy sources adopted for this study were coal, coke, gasoline, kerosene, diesel oil, fuel oil, liquefied petroleum gas, and natural gas. Next, the reference model provided by the IPCC was used to calculate the carbon emission of energy consumption [37], given by this formula:

(2) $$E_{b}=\partial \sum \left(EC_{i}\times NCV_{i}\times CEF_{i}\times OCF_{i}\right)$$

where, $E_{b}$ denotes the CO_2_ emissions from energy consumption; ∂ is the carbon content conversion coefficient, whose reference value is (44/12) [37]; $EC_{i}$ is the consumption of a certain energy source; $NCV_{i}$ is the net heat value of a certain energy source; $CEF_{i}$ is the carbon emission coefficient of a certain energy source; and $OCF_{i}$ is the oxidation coefficient of a certain energy source. In Table 2 are the reference values used for net heat value, carbon emission coefficient, and oxidation coefficient of the various energy sources.

Then, we calculated the carbon emission caused by construction land within the four basins by applying this formula:

(3) $$E_{sub-i}=T_{sub-i}\cdot \bar{\delta }=T_{sub-i}\cdot (E_{b}/T)$$

where, $E_{sub-i}$ denotes the carbon emission caused by construction land in a certain basin; $T_{sub-i}$ is the area of construction land in a certain basin; $\bar{\delta }$ is the average value of the carbon emission coefficient caused by construction land in Henan Province; $E_{b}$ is CO_2_ emissions from energy consumption, as calculated by Formula (2); and T is the total area of construction land in Henan Province.

(4) $$E_{sub}=E_{b}+E_{sub-i}$$

where, $E_{sub}$ is the total carbon emission caused by farmland and construction land in a certain basin.

The statistical analysis and spatial visualization of changes in carbon storage in the four basins were carried out in the R computing platform and ArcGis 10.7.

## 3. Results

### 3.1. Spatio-Temporal Variation of LUCC from 1990 to 2018

**Figure 2:**
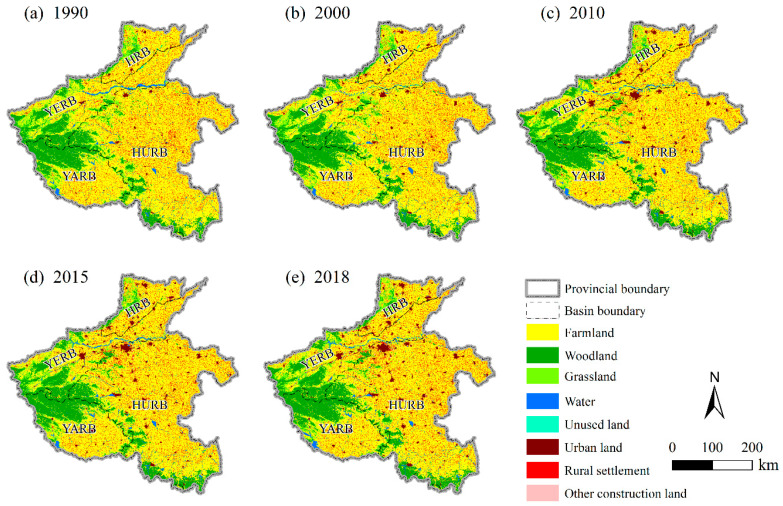
Land-use status in 1990 (**a**), 2000 (**b**), 2010 (**c**), 2015 (**d**), and 2018 (**e**) in Henan Province.

The spatial distributions of land use data in 1990, 2000, 2010, 2015, and 2018 showed that farmland is the main land-use type in Henan Province (Figure 2). Most farmland were found in the HURB. Ecological conservation land, such as woodland and grassland, were mainly distributed in the YERB and YARB. Significant spatial expansion of urban land has occurred in the HRB, YERB, and HURB. We calculated statistics on land use in the four basins according to land functions, to gauge the land-use conditions of the four basins in different periods (Table 3). The areas of food security land in the four river basins had this rank order: HURB > YERB > YARB > HRB. For ecological conservation land, YERB had the largest area, while HRB had the smallest area. In terms of production and living land, its area was still greater in the HURB than the other three basins. More detailed information on the respective area and proportion of land-use types (farmland, woodland, grassland, water, unused land, urban land, rural settlement, and other construction land) in different periods within the river basins can be found in Supplementary Table S1.

From the quantity and proportion of changes in land-use types (Table 4), the area of food security land declined in both the HRB and HURB during the five periods. In the YERB and YARB, the absolute area of food security land increased from 1990 to 2000, but it has decreased since 2000. Overall, from 1990 to 2018, HURB underwent the largest reduction in the area of food security land (−2761.50 km^2^), whereas it reduced least in the YERB (−256.76 km^2^). When consider on a proportional basis, the loss in area of food security land in HRB (−5.54%) exceeded that of the other three basins. Some differences among the four basins were evident in the change trend of ecological conservation land. The area decreased in every basin from 1990 to 2000, and it increased from 2010 to 2015. From 1990 to 2018, the area of ecological conservation land in the YARB increased by 150.46 km^2^ while in the other three basins it decreased (most pronounced in the YERB, at −967.22 km^2^). From 1990 to 2018, 3079.16 km^2^ of production and living land in HURB derived from another land use, an area significantly larger than that in the other three basins. By contrast, relatively small changes could be detected in the area of production and living land in the YARB. More detailed information on the quantity and proportion of changed land-use types (farmland, woodland, grassland, water, unused land, urban land, rural settlement, and other construction land) in the different periods within the four basins can be found in Supplementary Table S2.

**Figure 3:**
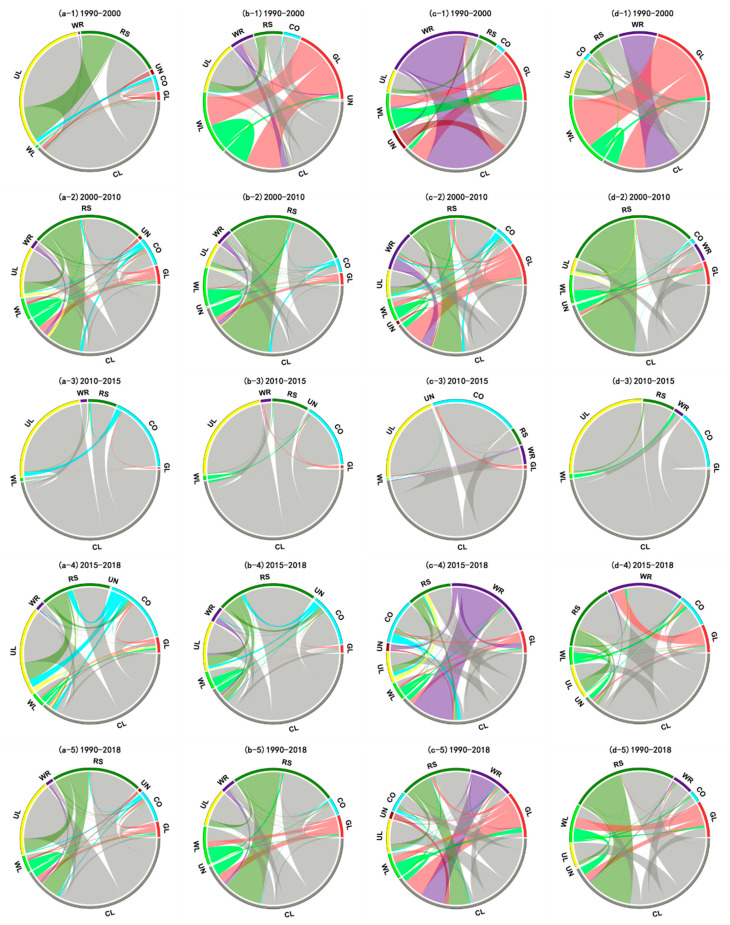
Chord diagrams of quantitative changes in land use/cover (LUCC) change in the four basins, from 1990 to 2018. (**a-1**), (**a-2**), (**a-3**), (**a-4**), and (**a-5**) show quantitative changes in LUCC in the HRB. (**b-1**), (**b-2**), (**b-3**), (**b-4**), and (**b-5**) show quantitative changes of LUCC in the HURB. (**c-1**), (**c-2**), (**c-3**), (**c-4**), and (**c-5**) show quantitative changes of LUCC in the YERB. (**d-1**), (**d-2**), (**d-3**), (**d-4**), and (**d-5**) show quantitative changes of LUCC in the YARB. WL, GL, WR, UN, CL, UL, RS, and CO denote the woodland, grassland, water, unused land, cultivated land, urban land, rural settlement, and other construction land, respectively.

We then implemented chord diagram visualizations to express the quantitative transformation relationships among land-use types (based on their transfer matrix), as they can more clearly describe the flow, direction, and diversity of LUCC. Evidently (Figure 3), from 1990 to 2000, the transformation from farmland to urban land played a leading role in the HRB (Figure 3(a-1)); in HURB, the outflows of grassland and farmland showed considerable dominance, in which grassland was mainly transformed into woodland and farmland, while farmland was converted to urban land use (Figure 3(b-1)), which contrasted with the YERB, where the transformation from water to farmland was more pronounced than that among other land-use types (Figure 3(c-1)); for the YARB, the transformations from grassland to woodland, water to farmland, and farmland to construction land were all prevalent forms of land-use change there (Figure 3(d-1)).

**Figure 4:**
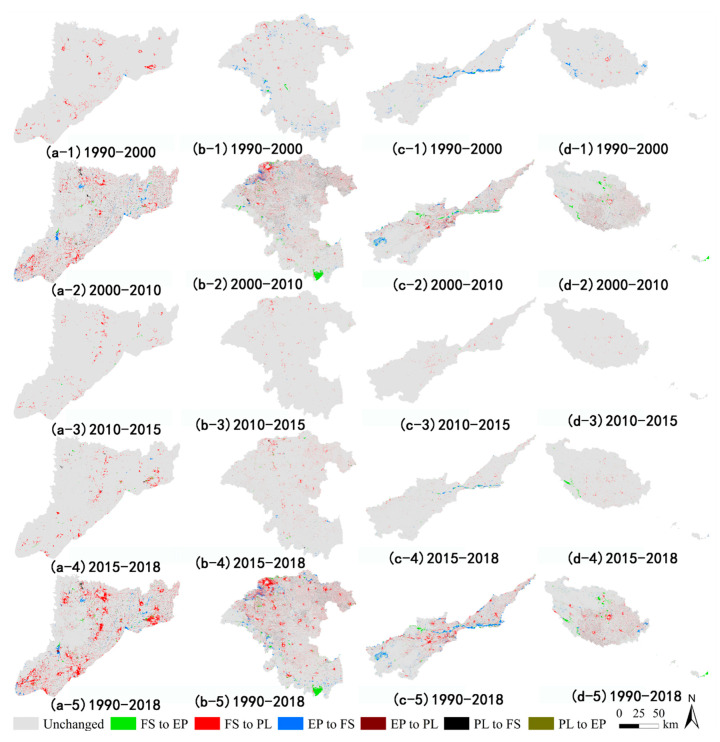
Spatial dynamics of land use/cover (LUCC) in the four basins from 1990 to 2018. (**a-1**), (**a-2**), (**a-3**), (**a-4**), and (**a-5**) depict spatial dynamics of LUCC in the HRB; (**b-1**), (**b-2**), (**b-3**), (**b-4**), and (**b-5**) depict spatial dynamics of LUCC in the HURB; (**c-1**), (**c-2**), (**c-3**), (**c-4**), and (**c-5**) depict spatial dynamics of LUCC in the YERB; (**d-1**), (**d-2**), (**d-3**), (**d-4**), and (**d-5**) depict spatial dynamics of LUCC in the YARB. The definition of land-use type abbreviations (FS, EP, PL) can be found in Table 4.

From 2000 to 2010, the reciprocal transformation between farmland and rural settlement predominated in all four basins (Figure 3(a-2)–(d-2)). Later, from 2010 to 2015, the LUCC in the four basins are all manifested in the transformation from farmland to urban land, rural settlement or other construction land (Figure 3(a-3)–(d-3)). Compared with the previous period, the diversity of land-use change in the four basins was greater between 2015 and 2018. For the HRB and HURB, farmland was mainly transformed into urban land, rural settlement, and other construction land (Figure 3(a-4),(b-4)), while the conversion of water to farmland predominated in the YERB (Figure 3(c-4)). In the YARB, the main land-use change was the transformation from farmland to water and rural settlement (Figure 3(d-4)).

**Figure 5:**
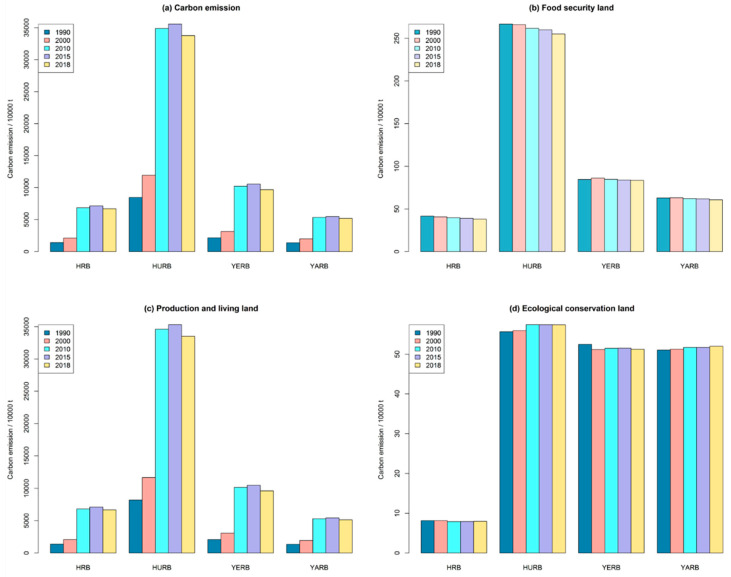
Quantity of carbon storage change in the study area in 1990, 2000, 2010, 2015, and 2018: (**a**) Total quantity of carbon emission; (**b**) Quantity of carbon emission from food security land; (**c**) Quantity of carbon emission from production and living land; (**d**) Quantity of carbon emission from ecological conservation land.

On the whole, from 1990 to 2018, the intensity of human activities in the four basins shifted; hence, there were certain differences in how land use had changed over time. These land-use changes in HRB and HURB (Figure 3(a-5)),(b-5)) were dominated by the conversion of food security land (farmland) to production and living land (urban land and rural settlement). Meanwhile, the transformation from production and living land (rural settlement) to food security land (farmland) in these two basins also warrants attention. In the YERB, its food security land (farmland) mainly flowed into production and living land, while its ecological conservation land (grassland and water) and production and living land (rural settlement) mainly became farmland (Figure 3(c-5)). The reciprocal transformation between food security land (farmland) and production and living land (rural settlement) was the main form of changed land use in the YARB (Figure 3(d-5)).

However, such quantitative changes cannot truly reflect the dynamic evolution of land-use change [38], because when a certain land-use type is converted to another in the same space, other types may revert to this land type in another place. Therefore, it is possible that no net changes in the quantity of a given land-use type occur over a certain period [39]. Accordingly, we used the overlay analysis function of ArcGIS 10.7 to visualize the spatial changes among land-use types, to better discern the actual process of land-use changes. As seen in Figure 4, the most dramatic changes in the spatial location of land use in the four basins occurred during the 2000–2010 period. In particular, the spatial positioning changes in the four basins were still significant during 2015–2018, despite only three years having passed.

### 3.2. Spatio-Temporal Variation of Land-Use-Related Carbon Storage Change from 1990 to 2018

According to the calculated carbon absorption results (Table 5), the size of the carbon sink in Henan Province was relatively stable, at 1.67–1.69 million tons, between 1990 and 2018, whereas its carbon emissions from carbon sources changed significantly during the five periods. Carbon emissions in 1990 and 2000 were 134.46 million tons and 191.79 million tons, respectively, but they were much higher in 2010, 2015, and 2018 at 573.06 million tons, 587.45 million tons, and 553.58 million tons respectively, a substantial increase over the previous two periods. In terms of the contribution of land use to carbon emissions, food security land in Henan Province was responsible for 4.56 million tons in 1990, which declined slightly to 4.37 million tons in 2018, in stark contrast to production and living land, which by far harbored the most carbon sources in Henan Province, making it the major contributor. As for ecological conservation land, it functioned as the only carbon sink contributor in Henan Province.

With respect to changes in carbon storage in the four basins, the carbon emitted from the HURB made the greatest contribution to Henan Province’s overall carbon emission (Figure 5a), because the former’s emissions caused by food security land and production and living land were greater than in other three basins, in five periods (Figure 5b,c). The YERB ranked second in terms of carbon emission, followed by the HRB and the YARB. Being the only carbon sink, carbon absorption by ecological conservation land in Henan Province mainly happened in the HURB, the YERB, and the YARB (Figure 5d). In summary, carbon absorption of the four basins was relatively stable during the five studied temporal periods, but their carbon emissions have increased significantly since 2010.

**Figure 6:**
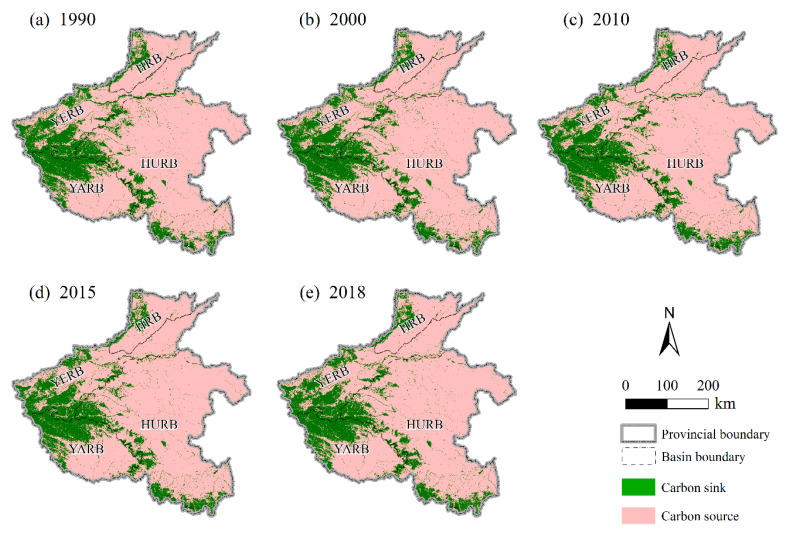
Spatial distribution of carbon sinks and carbon sources in the study area in 1990, 2000, 2010, 2015, and 2018.

As Figure 6 shows, the spatial distribution pattern of carbon sources and carbon sinks in Henan Province was consistent with the land-use pattern there. The carbon sink in Henan Province was mainly concentrated in the YARB and the YERB, which was conducive to the agglomeration effect of carbon absorption. Although the total amount of carbon absorption in the HURB was relatively large, the spatial distribution of its carbon sink was relatively scattered, resulting in a weaker carbon absorption effect. A strong carbon emission agglomeration effect was present in the HURB, so the pressure on ecological environment protection was also greater. Overall, no major changes occurred in the spatial distribution of carbon sources and carbon sinks in the four basins from 1990 to 2018.

## 4. Discussion

### 4.1. Advantages to Investigating Land-Use Change in Provincial Administrative Regions by Taking a River Basin Perspective

The four basins used in this paper were demarcated by their natural boundaries and the administrative boundary of Henan Province. Through a historical (retrospective) analysis of how land use has changed in Henan Province from 1990 to 2018, at the basin scale, we gleaned some interesting findings. Applying this perspective of basins lets us clearly understand the respective contribution made by the HRB, the HURB, the YERB, and the YARB to land-use dynamics in Henan Province. The HURB, having the largest land-use area among the four basins, contains the most food security land and production and living land. The YERB and the YARB are the ecological barriers of Henan Province with the largest area and concentrated spatial distribution of ecological conservation land. The land area of the HRB is the smallest among the four basins.

The flow, direction, and spatial patterning of reciprocal transformations among land types (Figure 3 and Figure 4) revealed that the 2000–2010 period was the most pivotal for land-use change in Henan Province. During this period, the conversion between farmland and construction land was a remarkable feature of four basins. From the late 1990s to the early 21st century, Henan Province has experienced a critical period of economic and social transformation [40]. A series of policies, such as “Construction of the Eastern New District of Zhengzhou,” the “Central Plains City Group,” and “Zheng-Bian integration” strategies, have rapidly promoted the economic restructuring of Henan Province. The development of its social economy will inevitably lead to the occupation of farmland by construction land [41]. Furthermore, in the late 1990s, the Chinese government implemented the most stringent farmland protection system to date. Key policies, namely, “Rural Settlement Renovation,” “Farmland Requisition and Compensation Balance,” and “Basic Farmland Protection” were successively implemented. Therefore, during this period, many rural settlements with unscientific overall arrangement were converted into farmland [42]. It is worth noting that the YARB was the only one of the four basins whose area of ecological conservation land had increased from 1990 to 2018.

In short, our results are more conducive to providing empirical data for the comprehensive governance of basins within a provincial administration, compared with the published results from the administrative regions at every level [43,44] or the main functional zones [42].

### 4.2. Analysis of Carbon Storage Changes Caused by Land-Use Changes

When the carbon sink is greater than the carbon source, we reasonably argued that the region in question is in a carbon surplus state; conversely, when this sink-to-source ration is reversed, the region is in a carbon deficit state [37]. Our present results revealed that Henan Province was in a state of carbon deficit from 1990 to 2018, which agrees with other recent findings [24,37]. In terms of the carbon storage changes in the four basins, the carbon emission from the HURB ranked first because it had the largest land expanse for food security and production and living. According to the carbon emission coefficients in Table 1, we find that woodland had the strongest carbon absorption capacity; hence, the HURB had the greatest carbon absorption (Figure 5d) due to its huge area of woodland. Further, we found the carbon emission caused by production and living land in 2018 was lower than that in 2015 (Figure 5c), perhaps because of improvements in energy consumption efficiency driven by the innovation of industrial production technology [18]. Importantly, we want to emphasize that, because of the strong carbon emission agglomeration effect of the HURB, Henan Province will likely face difficulty in becoming carbon neutral.

### 4.3. Suggestions for Carbon Emission Reduction under Land Use Transition

**Figure 7:**
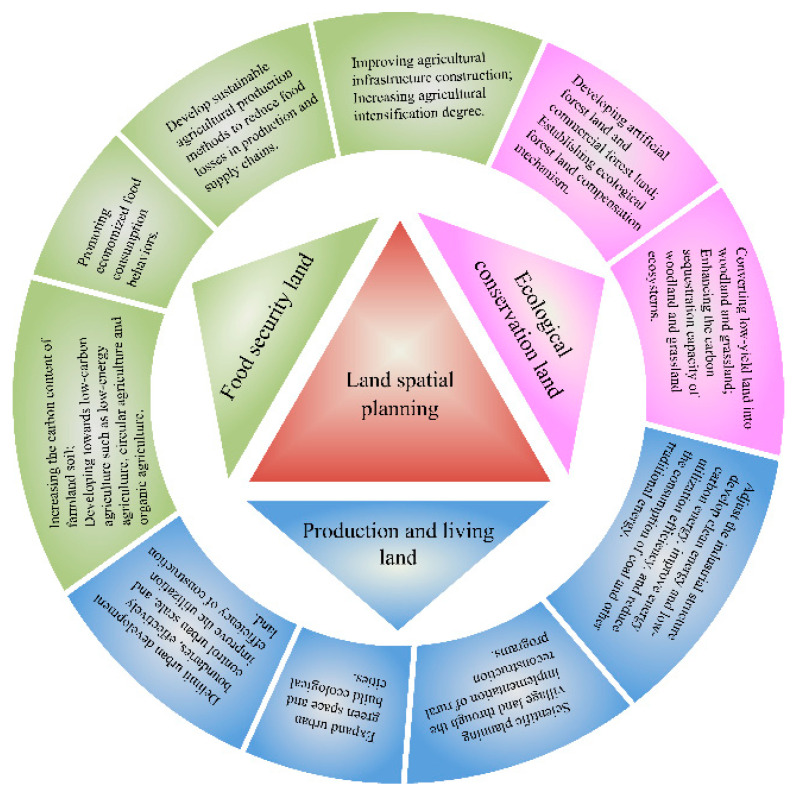
Suggestions for emission reduction based on land-use-related carbon storage changes.

Currently, the delimitation of an “Ecological Protection Red Line,” “Permanent Basic Farmland Protection Red Line,” and “Urban Development Boundary” are also important components in the spatial planning of China’s landscape. Therefore, we argue that the scientific overall arrangement of food security land, ecological conservation land, and production and living land should be rooted in the premise of a leading role for land spatial planning. Based on our research, we first put forward suggestions to reduce carbon emissions from the perspective of land-use changes (Figure 7). Then, we further discuss promising future efforts to reduce carbon emissions in the four basins in Henan Province.

Regarding how to achieve sustainable carbon emission reduction based on moderated land-use structure and improved land-use management: for food security land, we believe that the scale and planting intensity should be reasonably controlled while still meeting per capita food consumption demand and ensuring national food security. Therefore, the following four aspects merit special attention: (1) Figure 3 showed that the expansion of woodland and grassland to cultivated land from 1990 to 2018 was a common characteristic of the four watersheds. Therefore, it is urgent to find a way to reduce the need to transform additional land from a natural ecosystem into agricultural land. Improving agricultural infrastructure construction and increasing the degree of agricultural intensification can improve the production capacity of cultivated land, so as to prevent the excessive expansion of land resources [23]. (2) Improving agricultural production technology and reducing food loss during the circulation of agricultural products can effectively save cultivated land. (3) Population growth and food waste will lead to an increase in food demand, which will result in an expansion of cultivated land. Thus, economical food consumption behaviors ought to be promoted [23]. (4) It is important to increase farmland soil’s carbon content [45] and guide agriculture towards developing and implementing low-carbon agriculture, namely, low-energy, circular, and organic agriculture modes [9].

For ecological conservation land, we believe its ecological benefits could be improved in two ways: (1) First, by promoting the “Grain for Green Program (GGP)” policy, to convert more low-yield land (such as sloping fields, barren hills, and wasteland) into woodland and grassland, thereby enhancing the carbon sequestration capacity of these two ecosystems [12]. (2) Economic compensation is usually an effective measure to encourage people to carry out ecological protection. Hence, we suggested that an ecological compensation mechanism designed to encourage people to plant artificial and commercial forests should be established.

For production and living land, we propose the following four ways to control its carbon emissions: (1) delimit urban development boundaries, effectively control urban scale, and improve the utilization efficiency of construction land [46]; (2) expand urban green space and build ecological cities through the construction of urban forests, urban grasslands, street trees, parks, and urban wetlands [47]; (3) scientific planning of village land, via the implementation of rural reconstruction programs, such as “Demolition and Consolidation of Villages” and “Old Village Reconstruction” among others [28]; (4) adjust the industrial structure, develop clean energy and low-carbon energy, improve energy utilization efficiency, and reduce the consumption of coal and other traditional sources of energy [37].

Considering the contribution that the four river basins of Henan Province should make in the future: on the basis of the above suggestions, the four basins within Henan Province should play the following roles in multi-river basins comprehensive governance for the purpose of carbon emission reduction in the future.

For the YERB and the YARB: (1) Ecological resources are the advantages of the YERB and the YARB. We argued that forest tourism characteristic villages should be created relying on forest resources and water resources. (2) Develop characteristic economic gardens with flowers and seedlings to increase the value of agricultural output while increasing the carbon sequestration capacity of farmland ecosystem. (3) Accelerating the construction of ecological corridors in the YERB and the YARB by effective implementation of “Grain for Green Program.” Enhance ecological functions such as water conservation, soil erosion control, wind prevention and sand fixation, and actively build a composite ecological corridor integrating ecological protection, cultural promotion, leisure and tourism, and ecological agriculture.

For the HRB and the HURB: The rapid expansion of production and living land is a common characteristic of the two river basins. Therefore, the construction of an ecological city should be determined as the primary goal of the HRB and the HURB to enhance the ecological effect of urban green space as the “kidney of the city.” For doing this, the city scale should be controlled reasonably, and the spatial configuration relationship between urban buildings and urban green space should be considered comprehensively.

## 5. Conclusions

Assessing the carbon emission effects caused by LUCC from a river basin perspective instead of an administrative region boundary offers a new approach for the comprehensive governance of river basin, based on ecological protection at the provincial administrative level. Firstly, we reviewed the temporal and spatial evolution characteristics of land-use change in the HRB, the YERB, the HURB, and the YARB in Henan Province, in 1990, 2000, 2010, 2015, and 2018. Then, we estimated carbon storage changes caused by land-use changes in the four basins. Finally, we put forward evidence-based suggestions to enhance their comprehensive governance in the future, with a view towards attaining the complementary goals of energy conservation and emission reduction.

From 1990 to 2018, the amount of food security land decreased in every basin within Henan Province, being reduced most in the HURB (a decrease of 2761.50 km^2^) and least in the YERB (a decrease of 256.76 km^2^). The ecological conservation land in the YARB has increased by 150.46 km^2^ in the past 28 years, but this land use declined in the other three basins, with the largest decrease found in the YERB (a decrease of 967.22 km^2^). From 1990 to 2018, the area of production and living land in increased in all four basins, by 3079.16 km^2^ in the HURB, which exceeded the gains made in the other three basins. Nonetheless, transforming what was once food security land into production and living land was a distinguishing feature common to all four basins. Further, the changes in the spatial location of land use in these four basins from 2000 to 2010 could better represent the overall change trend of the past 29 years.

From 1990 to 2018, Henan Province’s carbon emission increased from 134.46 to 553.58 million tons, while its carbon absorption increased from 1.67 to 1.69 million tons. Since 1990, Henan Province has been in a state of carbon deficit, a situation that deteriorated further after 2010. With the most farmland and construction land, the carbon emission of the HURB was the main contributor to carbon emissions of Henan Province, whose main carbon sink areas were ecological conservation land in the HURB, the YERB, and the YARB. Spatially, however, carbon absorption in the YERB and the YARB showed clear signs of an agglomeration effect.

To achieve the goal of carbon neutrality by 2060 as set by the Chinese government, we suggest that carbon sequestration capacity should be further strengthened in the YERB and the YARB based on their respective ecological resource advantages in terms of woodland, grassland, and water resources. For future development in the HRB and the HURB, coordinating the spatial allocation of urban scale and urban green space to build an ecological city is a key direction to embark upon.

## Figures and Tables

**Table 1:** Emission (absorption) coefficient of the five types of land use studied in Henan.

Land-Use Types	Emission (Absorption) Coefficient	Unit
Woodland	−0.00578	kt/(km^2^·a)
Grassland	−0.00021	kt/(km^2^·a)
Water	−0.00252	kt/(km^2^·a)
Unused land	−0.00005	kt/(km^2^·a)
Farmland	0.00422	kt/(km^2^·a)

Notes: A negative value in this table denotes the carbon *absorption* coefficient, and a positive value indicates the carbon *emission* coefficient. Table 1 draws from the results of Cao and Yuan [5] and Xu et al. [34].

**Table 2:** Net heat value, carbon emission coefficient, and oxidation coefficient of the eight energy sources.

Item	Coal	Coke	Gasoline	Kerosene	Diesel Oil	Fuel Oil	Liquefied Petroleum Gas	Natural Gas
Net heat value (TJ/kt)	20.93	28.47	43.12	44.10	42.71	41.87	47.47	38.90
Carbon emission coefficient (t/TJ)	26.80	29.41	18.90	19.60	20.17	21.09	17.20	15.32
Oxidation coefficient (%)	91.50	92.80	98.00	98.60	98.20	98.50	98.50	99.00

Notes: Table 2 draws from the results of Wang [37].

**Table 3:** Area and proportion of land-use types in the study area in 1990, 2000, 2010, 2015, and 2018.

Region	Land Use Type	1990		2000		2010		2015		2018	
		AR (km^2^)	PR (%)	AR (km^2^)	PR (%)	AR (km^2^)	PR (%)	AR (km^2^)	PR (%)	AR (km^2^)	PR (%)
HRB	FS	9861.13	66.03	9674.89	64.78	9388.57	62.87	9251.77	61.95	9033.73	60.49
	EC	3361.42	22.51	3354.03	22.46	3242.63	21.71	3247.96	21.75	3262.80	21.85
	PL	1711.79	11.46	1905.44	12.76	2303.14	15.42	2434.61	16.30	2637.79	17.66
	Total	14,934.34	100	14,934.36	100	14,934.34	100	14,934.34	100	14,934.32	100
HURB	FS	63,195.49	73.19	63,030.26	73.00	62,009.76	71.81	61,609.66	71.35	60,434.00	69.99
	EC	12,987.47	15.04	12,532.53	14.51	12,636.28	14.63	12,639.31	14.64	12,670.76	14.67
	PL	10,162.57	11.77	10,783.99	12.49	11,700.71	13.55	12,097.76	14.01	13,241.73	15.34
	Total	86,345.53	100	86,346.78	100	86,346.75	99.99	86,346.73	100	86,346.49	100
YERB	FS	20,043.68	54.86	20,438.80	55.94	20,082.97	54.96	19,917.28	54.51	19,786.92	54.15
	EC	13,920.89	38.10	13,279.31	36.34	13,025.69	35.65	13,033.42	35.67	12,953.67	35.45
	PL	2573.08	7.04	2819.59	7.72	3429.09	9.39	3587.05	9.82	3797.14	10.39
	Total	36,537.65	100	36,537.7	100	36,537.75	100	36,537.75	100	36,537.73	99.99
YARB	FS	14,914.11	54.05	14,958.95	54.22	14,734.26	53.40	14,665.78	53.15	14,390.52	52.16
	EC	11,019.25	39.94	10,842.98	39.30	11,066.52	40.11	11,068.38	40.11	11,169.71	40.48
	PL	1658.46	6.01	1789.89	6.49	1791.03	6.49	1857.65	6.73	2031.55	7.36
	Total	27,591.82	100	27,591.82	100	27,591.81	100	27,591.81	99.99	27,591.78	100

Note: ① “FS” denotes “Food security land”; “EC” denotes “Ecological conservation land”; “PL” denotes “Production and living land”. ② “AR” denotes “Area”; “PR” denotes “Proportion”. HRB, Hai River Basin; HURB, Huai River Basin; YERB, Yellow River Basin; YARB, Yangtze River Basin.

**Table 4:** Quantity and proportion of changes to types of land use in the study area during 1990–2018.

Region	Land Use Type	1990–2000		2000–2010		2010–2015		2015–2018		1990–2018	
		AR (km^2^)	PR (%)	AR (km^2^)	PR (%)	AR (km^2^)	PR (%)	AR (km^2^)	PR (%)	AR (km^2^)	PR (%)
HRB	FS	−186.23	−1.25	−286.32	−1.92	−136.80	−0.92	−218.04	−1.46	−827.39	−5.54
	EC	−7.39	−0.05	−111.41	−0.75	5.33	0.04	14.84	0.10	−98.63	−0.66
	PL	193.65	1.30	397.71	2.66	131.47	0.59	203.18	1.36	926.00	6.20
HURB	FS	−165.23	−0.19	−1020.51	−1.18	−400.09	−0.46	−1175.66	−1.36	−2761.50	−3.20
	EC	−454.94	−0.53	103.74	0.12	3.04	0.00	31.44	0.04	−316.72	−0.37
	PL	621.42	0.72	916.72	1.06	397.05	0.46	1143.97	1.32	3079.16	3.57
YERB	FS	395.13	1.08	−355.83	−0.97	−165.69	−0.45	−130.36	−0.36	−256.76	−0.70
	EC	−641.57	−1.76	−253.62	−0.69	7.73	0.02	−79.75	−0.22	−967.22	−2.65
	PL	246.51	0.67	609.49	1.67	157.96	0.43	210.09	0.58	1224.06	3.35
YARB	FS	44.84	0.16	−224.69	−0.81	−68.48	−0.25	−275.26	−1.00	−523.59	−1.90
	EC	−176.26	−0.64	223.53	0.81	1.86	0.01	101.33	0.37	150.46	0.55
	PL	131.43	0.48	1.14	0.00	66.62	0.24	173.90	0.63	373.09	1.35

Notes: ① “FS” denotes “Food security land”; “EC” denotes “Ecological conservation land”; “PL” denotes “Production and living land”. ② “AR” denotes “Area”; “PR” denotes “Proportion”.

**Table 5:** Change in carbon storage in Henan Province, from 1990 to 2018.

Item	1990	2000	2010	2015	2018
Food security land (million tons)	4.56	4.56	4.48	4.45	4.37
Ecological conservation land (million tons)	−1.67	−1.67	−1.69	−1.69	−1.69
Production and living land (million tons)	129.91	187.23	568.57	583.00	549.21
Carbon source (million tons)	134.46	191.79	573.06	587.45	553.58
Carbon sink (million tons)	−1.67	−1.67	−1.69	−1.69	−1.69

## Data Availability

Publicly available datasets were analyzed in this study. This data can be found here: Henan Statistical Yearbook (https://navi.cnki.net/KNavi/YearbookDetail?pcode=CYFD&pykm=YHNJT&bh=), SRTMDEMUTM 90M Resolution Digital Elevation Data (http://www.gscloud.cn/search), Resource and Environment Science and Data Center of the Chinese Academy of Sciences (http://www.resdc.cn/data.aspx?DATAID=200), China Energy Statistical Yearbook (https://navi.cnki.net/KNavi/YearbookDetail?pcode=CYFD&pykm=YCXME&bh=).

## Supplementary Materials

The following are available online at https://www.mdpi.com/1660-4601/18/4/1844/s1. Table S1: Area and proportion of the land use types of the study area in 1990, 2000, 2010, 2015, and 2018. Table S2: Quantity and proportion of changes of the land use types of the study area in 1990–2018.
