# Supplementary material for: Carbon Storage Change Analysis and Emission Reduction Suggestions under Land Use Transition: A Case Study of Henan Province, China
Source: Int J Environ Res Public Health. 2021 Feb 14;18(4):1844. doi: 10.3390/ijerph18041844 (PMC7918624; doi:10.3390/ijerph18041844)
Supplement: Supplementary file 1 [file ijerph-18-01844-s001.pdf]

Supplementary table S1. Area and proportion of the land use types of the study area in 1990, 2000, 2010, 2015, and 2018.

Region	Land use Type		1990		2000		2010		2015		2018	
			Area/km^2	Proportion/%	Area/km^2	Proportion/%	Area/km^2	Proportion/%	Area/km^2	Proportion/%	Area/km^2	Proportion/%
HRB	Food security land	Farmland	9861.13	66.03	9674.89	64.78	9388.57	62.87	9251.77	61.95	9033.73	60.49
	Ecological conservation land	Woodland	1281.43	8.58	1281.25	8.58	1244.24	8.33	1246.25	8.34	1249.41	8.37
		Grassland	1920.05	12.86	1919.16	12.85	1872.81	12.54	1871.92	12.53	1874.61	12.55
		Water	132.82	0.89	132.62	0.89	124.07	0.83	128.28	0.86	137.28	0.92
		Unused land	27.12	0.18	21.00	0.14	1.50	0.01	1.50	0.01	1.50	0.01
	Production and living land	Urban land	308.92	2.07	482.09	3.23	722.68	4.84	793.33	5.31	920.21	6.16
		Rural settlement	1334.85	8.94	1345.63	9.01	1428.03	9.56	1445.26	9.68	1475.53	9.88
		Other construction land	68.02	0.46	77.72	0.52	152.43	1.02	196.02	1.31	242.06	1.62
HURB	Food security land	Farmland	63195.49	73.19	63030.26	73.00	62009.76	71.81	61609.66	71.35	60434.00	69.99
	Ecological conservation land	Woodland	8768.31	10.15	8819.80	10.21	9027.16	10.45	9018.55	10.44	9006.28	10.43
		Grassland	2423.71	2.81	1881.01	2.18	1654.02	1.92	1647.94	1.91	1668.91	1.93
		Water	1788.62	2.07	1826.00	2.11	1953.43	2.26	1970.62	2.28	1992.71	2.31
		Unused land	6.83	0.01	5.73	0.01	1.66	0.00	2.20	0.00	2.86	0.00
	Production and living land	Urban land	718.40	0.83	1162.14	1.35	2022.39	2.34	2221.14	2.57	2611.18	3.02
		Rural settlement	9322.49	10.80	9372.23	10.85	9297.06	10.77	9367.28	10.85	9823.81	11.38
		Other construction land	121.68	0.14	249.62	0.29	381.26	0.44	509.34	0.59	806.74	0.93
YERB	Food security land	Farmland	20043.68	54.86	20438.80	55.94	20082.97	54.96	19917.28	54.51	19786.92	54.15
	Ecological conservation land	Woodland	8331.05	22.80	8299.92	22.72	8272.24	22.64	8271.66	22.64	8264.48	22.62
		Grassland	4073.79	11.15	3983.76	10.90	3577.08	9.79	3574.30	9.78	3574.99	9.78
		Water	1376.69	3.77	936.73	2.56	1162.56	3.18	1173.56	3.21	1089.05	2.98
		Unused land	139.35	0.38	58.90	0.16	13.81	0.04	13.90	0.04	25.15	0.07
	Production and living land	Urban land	209.33	0.57	350.31	0.96	751.85	2.06	824.97	2.26	889.75	2.44
		Rural settlement	2250.81	6.16	2305.45	6.31	2452.42	6.71	2466.32	6.75	2525.74	6.91
		Other construction land	112.94	0.31	163.83	0.45	224.81	0.62	295.75	0.81	381.64	1.04
YARB	Food security land	Farmland	14914.11	54.05	14958.95	54.22	14734.26	53.40	14665.78	53.15	14390.5204	52.16
	Ecological conservation land	Woodland	8455.38	30.64	8536.92	30.94	8563.72	31.04	8562.37	31.03	8549.8052	30.99
		Grassland	1854.91	6.72	1678.66	6.08	1765.20	6.40	1765.21	6.40	1738.5799	6.30
		Water	708.95	2.57	627.40	2.27	736.97	2.67	740.17	2.68	880.6556	3.19
		Unused land	0.00	0.00	0.00	0.00	0.62	0.00	0.62	0.00	0.6684	0.00
	Production and living land	Urban land	86.51	0.31	167.10	0.61	233.55	0.85	271.62	0.98	328.416	1.19
		Rural settlement	1561.91	5.66	1597.28	5.79	1520.74	5.51	1530.09	5.55	1589.5856	5.76
		Other construction land	10.05	0.04	25.51	0.09	36.74	0.13	55.94	0.20	113.5494	0.41

Supplementary table S2. Quantity and proportion of changes of the land use types of the study area in 1990-2018.

Region	Land use Type		1990–2000		2000–2010		2010–015		2015–2018		1990–2018	
			Area/km²	Proportion/%	Area/km²	Proportion/%	Area/km²	Proportion/%	Area/km²	Proportion/%	Area/km²	Proportion/%
HRB	Food security land	Farmland	−186.23	−1.25	−286.32	−1.92	−136.80	−0.92	−218.04	−1.46	−827.39	−5.54
	Ecological conservation land	Woodland	−0.18	0.00	−37.01	−0.25	2.01	0.01	3.16	0.02	−32.01	−0.21
		Grassland	−0.89	−0.01	−46.35	−0.31	−0.89	−0.01	2.69	0.02	−45.44	−0.30
		Water	−0.20	0.00	−8.55	−0.06	4.21	0.03	8.99	0.06	4.45	0.03
		Unused land	−6.12	−0.04	−19.50	−0.13	0.00	0.00	−0.01	0.00	−25.62	−0.17
	Production and living land	Urban land	173.17	1.16	240.59	1.61	70.65	0.47	126.88	0.85	611.29	4.09
		Rural settlement	10.77	0.07	82.40	0.55	17.23	0.12	30.27	0.20	140.68	0.94
		Other construction land	9.70	0.06	74.71	0.50	43.59	0.29	46.04	0.31	174.04	1.17
HURB	Food security land	Farmland	−165.23	−0.19	−1020.51	−1.18	−400.09	−0.46	−1175.66	−1.36	−2761.50	−3.20
	Ecological conservation land	Woodland	51.49	0.06	207.36	0.24	−8.61	−0.01	−12.27	−0.01	237.97	0.28
		Grassland	−542.70	−0.63	−226.99	−−0.26	−6.08	−0.01	20.97	0.02	−754.80	−0.87
		Water	37.37	0.04	127.44	0.15	17.19	0.02	22.09	0.03	204.09	0.24
		Unused land	−1.10	0.00	−4.07	0.00	0.54	0.00	0.66	0.00	−3.97	0.00
	Production and living land	Urban land	443.74	0.51	860.25	1.00	198.75	0.23	390.04	0.45	1892.78	2.19
		Rural settlement	49.74	0.06	−75.17	−0.09	70.22	0.08	456.53	0.53	501.32	0.58
		Other construction land	127.94	0.15	131.64	0.15	128.08	0.15	297.40	0.34	685.06	0.79
YERB	Food security land	Farmland	395.13	1.08	−355.83	−0.97	−165.69	−0.45	−130.36	−0.36	−256.76	−0.70
	Ecological conservation land	Woodland	−31.13	−0.09	−27.68	-0.08	-0.58	0.00	−7.18	−0.02	−66.57	−0.18
		Grassland	−90.03	−0.25	−406.68	−1.11	−2.78	−0.01	0.69	0.00	−498.80	−1.37
		Water	−439.95	−1.20	225.83	0.62	10.99	0.03	−84.50	−0.23	−287.63	−0.79
		Unused land	−80.46	−0.22	−45.09	−0.12	0.09	0.00	11.25	0.03	−114.21	−0.31
	Production and living land	Urban land	140.98	0.39	401.55	1.10	73.12	0.20	64.78	0.18	680.42	1.86
		Rural settlement	54.64	0.15	146.97	0.40	13.90	0.04	59.42	0.16	274.93	0.75
		Other construction land	50.90	0.14	60.98	0.17	70.94	0.19	85.89	0.24	268.71	0.74
YARB	Food security land	Farmland	44.84	0.16	−224.69	−0.81	−68.48	−0.25	−275.26	−1.00	−523.59	−1.90
	Ecological conservation land	Woodland	81.53	0.30	26.80	0.10	−1.34	0.00	−12.57	−0.05	94.42	0.34
		Grassland	−176.25	−0.64	86.54	0.31	0.01	0.00	−26.63	−0.10	−116.33	−0.42
		Water	−81.55	−0.30	109.57	0.40	3.19	0.01	140.49	0.51	171.70	0.62
		Unused land	0.00	0.00	0.62	0.00	0.00	0.00	0.04	0.00	0.67	0.00
	Production and living land	Urban land	80.60	0.29	66.45	0.24	38.07	0.14	56.80	0.21	241.91	0.88
		Rural settlement	35.37	0.13	−76.54	−0.28	9.35	0.03	59.49	0.22	27.68	0.10
		Other construction land	15.46	0.06	11.23	0.04	19.20	0.07	57.61	0.21	103.50	0.38
